# Causal association between circulating Klotho levels and B-cell lymphoma: A 2-sample Mendelian randomization study

**DOI:** 10.1097/MD.0000000000044963

**Published:** 2025-10-31

**Authors:** Zi Wang, Su Mao, Daobin Zhou, Wei Zhang

**Affiliations:** a Department of Hematology, Peking Union Medical College Hospital, Peking Union Medical College, Chinese Academy of Medical Sciences, Beijing, China; b Department of Obstetrics and Gynecology, National Clinical Research Center for Obstetric and Gynecologic Diseases, Peking Union Medical College Hospital, Peking Union Medical College, Chinese Academy of Medical Sciences, Beijing, China.

**Keywords:** B-cell lymphoma, diffuse large B-cell lymphoma, Klotho, Mendelian randomization, single nucleotide polymorphisms

## Abstract

This study aimed to investigate a causal association between Klotho levels and the risk of B-cell lymphoma using a Mendelian randomization (MR) approach. We sought to determine if α-Klotho levels are causally linked to various subtypes of B-cell lymphoma, including diffuse large B-cell lymphoma, follicular lymphoma, Hodgkin lymphoma, and non-Hodgkin lymphoma. We obtained GWAS data for B-cell lymphomas from the FINN Large Cohort in the IEU database and α-Klotho levels from a meta-analysis of GWAS data. The primary analysis employed the inverse-variance weighted method, while sensitivity analyses utilized weighted median, MR-Egger, and weighted mode methods to validate results. Heterogeneity and pleiotropy of genetic instruments were assessed using leave-one-out sensitivity tests, the MR pleiotropy residual sum and outlier test (MR-PRESSO), and Cochran *Q* test. Our analysis revealed no significant associations between α-Klotho levels and any subtype of B-cell lymphoma using inverse-variance weighted (IVW). The odds ratios and 95% confidence intervals indicated no significant relationship for diffuse large B-cell lymphoma, follicular lymphoma, Hodgkin lymphoma, or unspecified non-Hodgkin lymphoma. Heterogeneity tests and sensitivity analyses supported the robustness of these findings. Our comprehensive MR analysis suggests no causal relationship exists between Klotho levels and the risk of developing B-cell lymphomas.

## 1. Introduction

B-cell lymphoma is a type of non-Hodgkin lymphoma that starts in white B-cells. It is often found in lymph nodes or other lymphoid tissues, such as the spleen.^[1]^ Diffuse large B-cell lymphoma is the most common subtype that accounts for a third of all non-Hodgkin lymphomas, ranging between 20% and 50%, depending on the country.^[2]^ Other common subtypes include follicular lymphoma, a slow-growing (indolent) lymphoma, chronic lymphocytic leukemia/small lymphocytic lymphoma, Mantle cell lymphoma, marginal zone lymphoma, and Burkitt lymphoma.^[1]^ Regarding treatment, the majority of B-cell lymphoma patients respond to chemotherapy; yet, some may still present a refractory or relapsed process.^[3]^ Therefore, more effective treatment strategies based on molecular oncogenic pathways and novel therapeutic targets are still needed.

B-cell lymphoma affects any age but is predominantly seen in aged patients.^[1,4]^ It is well known that cancer and aging share comparable principles and that time-dependent accumulation of DNA damage and genetic defects is a contributing factor that drives cancer progression.^[5]^ Klotho is an antiaging protein predominantly produced in the kidney, with the amino-terminal extracellular domain shedding into the systemic circulation.^[6]^ This antiaging protein has diverse beneficial clinical implications.^[7]^ Besides being antiaging, it has health span and lifespan-extending, cognitive enhancing, anti-oxidative, anti-inflammatory, and antitumor properties.^[7]^ The Klotho gene is associated with an increased risk of age-related diseases, such as cognitive impairment, diminished longevity, and reduced growth.^[8]^ In addition, few studies have found that Klotho protein acts as a tumor suppressor and modulator of IGFR1 signaling in T-cell lymphoma^[9]^ and some types of B-cell lymphoma, such as diffuse large B-cell lymphoma.^[10]^ Yet, confounding factors in this study were not analyzed.

Mendelian randomization (MR) is a method based on Mendel first and second laws of genetic inheritance.^[11]^ A genetic variant is considered an instrumental variable (IV) for a given exposure if it satisfies the IV assumptions.^[12]^ So far, MR has been increasingly applied to examine causal inference in B-cell lymphoma.^[13–15]^ However, the relationship between Klotho protein and B-cell lymphoma using the MR method has not yet been investigated. In this study, we applied an MR analysis to investigate a causal association between α-Klotho protein and B-cell lymphoma.

## 2. Methods

### 2.1. Study design

**Figure 1.**
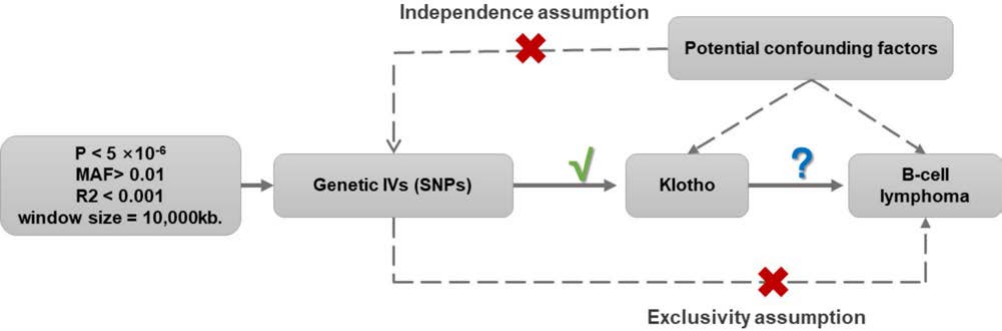
Overview of MR. MR = Mendelian randomization.

In our study, single nucleotide polymorphisms (SNPs) from Genome-wide association study (GWAS) were selected as genetic IVs. As illustrated in Figure 1, our 2-sample MR study was structured around 3 principal assumptions^[12]^: relevance assumption: The IVs demonstrated a strong association with the exposure. Independence assumption: There was no correlation between the IVs and any confounders that affected both the exposure and the outcome. Exclusion restriction assumption: The IVs influenced the outcome solely through their effects on the exposure, without any alternative causal pathways.

### 2.2. Ethics statement

According to Article 32 of the Ethical Review Measures for Life Science and Medical Research Involving Human Subjects of the People’s Republic of China, the database used in this study is publicly available and legally accessible. It does not cause any harm to humans or involve sensitive personal privacy or commercial secrets, and therefore qualifies for exemption from ethical review.

### 2.3. Data source

GWAS data for B-cell lymphomas were obtained from the FINN Large Cohort in the IEU database. Data for α-Klotho levels were sourced from a previous study,^[16]^ i.e., GWAS meta-analysis followed by MR that revealed potential control mechanisms for circulating α-Klotho levels. Detailed information on the sample sizes (N) and data sources for the GWAS studies is provided in Table S1, Supplemental Digital Content, https://links.lww.com/MD/Q357.

### 2.4. Selection of IVs

To ensure the selection of IVs that adhere to the core assumptions of MR, 5 critical steps of quality control were implemented^[17]^: Initial SNP Selection: SNPs with strong associations to the exposure were selected based on a stringent *P*-value <5 × 10^−8^.^[18,19]^ Minor allele frequency Check: SNPs with a minor allele frequency <0.01 were excluded to avoid potential biases caused by rare genetic variations.^[20]^ Linkage disequilibrium (LD) clumping: A clumping process was applied with an LD threshold of *R*^2^ <0.001 and a window size of 10,000kb were applied. This step was taken to eliminate SNPs in strong LD, thus reducing redundancy and potential confounding from closely linked genetic variants.^[21]^ Proxy SNP Selection: In instances where the targeted SNPs were not available in the GWAS summary data, proxy SNPs with a high LD (*R*^2^ >0.8) with the targeted SNPs were identified and used as replacements. This ensures that the analysis still captures the genetic variation intended for the study. Final SNP Validation: The *F* value for each SNP in the IV was used to evaluate the strength of the IV, excluding the possibility of weak instrument bias between the IV and the exposure factor. The following formula was applied for calculation: *F* = *r*^2^* (N − 2)/ (1 − r2). where *R*^2^ is the proportion of exposure variance explained by the SNP in the IV, and the requirement for the *F* value is >10.^[22]^

### 2.5. MR analysis

This study employed a variety of MR methods to ascertain the causal effects of exposure on outcomes. The primary method used was the inverse-variance weighted (IVW) approach, which provides a weighted average of Wald ratio estimates, assuming that IVs influence the outcome solely through the exposure.^[23]^ The MR-Egger regression offers a causal estimate that accounts for unbalanced pleiotropy by assuming the Instrument Strength Independent of Direct Effect (InSIDE) and including an intercept term, although it has lower precision and can be affected by outlying variants.^[24]^ The Weighted Median approach, assuming validity of at least 50% of the IVs, is less susceptible to horizontal pleiotropic bias,^[25]^ delivering a robust causal estimate. Additionally, the Weighted Mode method groups SNPs by similar causal effects for estimation.^[26]^

Causal associations were primarily analyzed using IVW and presented as odds ratios with 95% confidence intervals. MR-Egger, Weighted Median, and Weighted Mode analyses were also conducted to confirm the robustness of our findings. All analyses in this study were conducted using the “TwoSampleMR” package (R version 4.0.5). Visualizations were performed using scatter plots and sensitivity analysis plots. Due to the presence of 6 outcome factors in this study, the false discovery rate correction method was used to correct the P-values for multiple testing, with *P*_FDR_ <.05 considered statistically significant.

### 2.6. Sensitivity analysis

Cochran *Q* test^[27]^ was used to assess heterogeneity among IVs, with *P* >.05 indicating low heterogeneity, meaning the estimates among IVs were randomly distributed and had little impact on IVW results. MR-Egger regression method was applied to explore the presence of horizontal pleiotropy; when the intercept of MR-Egger regression approached zero or was not statistically significant, this suggested the absence of pleiotropy.^[28]^ Additionally, the MR pleiotropy residual sum and outlier (MR-PRESSO) method was used to detect potential outliers (i.e., SNPs with *P* <.05) and reestimate causal associations after their removal to correct for horizontal pleiotropy.^[29]^ Leave-one-out analysis was employed to assess the robustness and consistency of the results.^[30]^

## 3. Results

### 3.1. Selection of instrumental variables

In this study, IVs were selected based on circulating plasma levels of α-Klotho as the exposure factor, with B-cell lymphoma serving as the outcome. A total of 5 IVs were identified using genome-wide significant SNPs associated with α-Klotho levels. Detailed SNP information is provided in the supplementary file, Supplemental Digital Content, https://links.lww.com/MD/Q358. The selected IVs demonstrated a mean *F*-statistic of 71.93, with a range from 32.95 to 117.73, indicating strong instrument strength. All SNPs matched the information in the summary data, with an *R*^2^ value of 0.076, suggesting minimal bias due to weak instruments.^[16]^

### 3.2. MR analysis results

**Figure 2.**
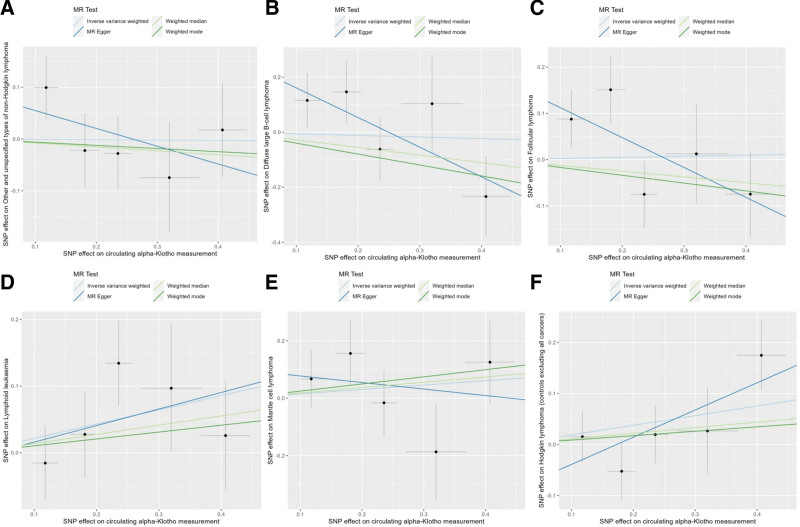
Scatter plots visualize the causal effects of Klotho levels on B-cell lymphoma. (A) Other and unspecified types of non-Hodgkin lymphoma (all cancers excluded); (B) diffuse large B-cell lymphoma (all cancers excluded); (C) follicular lymphoma (all cancers excluded); (D) lymphoid leukaemia; (E) Mantle cell lymphoma; (F) Hodgkin lymphoma (controls excluding all cancers).

**Figure 3.**
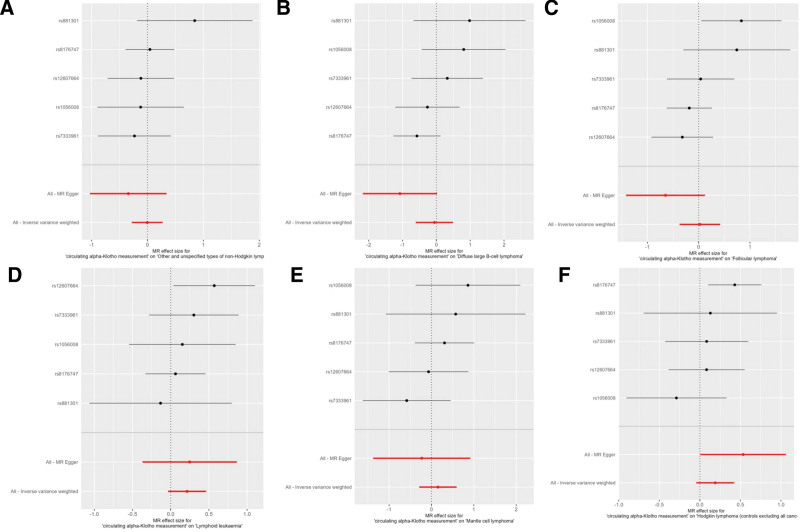
Forest plot demonstrating estimated effect size of associations between Klotho levels and B-cell lymphoma. (A) Other and unspecified types of non-Hodgkin lymphoma (all cancers excluded); (B) diffuse large B-cell lymphoma (all cancers excluded); (C) follicular lymphoma (all cancers excluded); (D) lymphoid leukaemia; E) Mantle cell lymphoma; (F) Hodgkin lymphoma (controls excluding all cancers).

The primary analysis using the IVW method found no statistically significant causal association between genetically predicted circulating α-Klotho levels and the risk of any of the 6 investigated subtypes of B-cell lymphoma. The detailed results are presented in Table 1. Specifically, no causal effect was observed for other and unspecified types of non-Hodgkin lymphoma (OR = 0.99, 95% CI: 0.75–1.31, *P* = .97), diffuse large B-cell lymphoma (OR = 0.94, 95% CI: 0.54–1.64, *P* = .84), or follicular lymphoma (OR = 1.02, 95% CI: 0.69–1.52, *P* = .91). Furthermore, there was no evidence of a causal relationship for lymphoid leukaemia (OR = 1.24, 95% CI: 0.97–1.59, *P* = .09), mantle cell lymphoma (OR = 1.17, 95% CI: 0.75–1.81, *P* = .50), or Hodgkin lymphoma (OR = 1.21, 95% CI: 0.96–1.53, *P* = .11). The MR-Egger, Weighted Median, and Weighted Mode methods, were consistent with the primary IVW results, further supporting the absence of a causal relationship across all lymphoma subtypes (Table 1). The scatter plots and forest plots visually represent these null findings (Figs. 2 and 3). Overall, the MR analyses collectively indicate that there is no significant causal relationship between circulating levels of α-Klotho and the risk of B-cell lymphoma across different analytical methods.

**Table 1:** Klotho levels and B-cell lymphoma in the MR analyses.

Exposure	Outcome	N. SNPs	Methods	OR (95% CI)	*P*
Circulating alpha-Klotho measurement	Other and unspecified types of non-Hodgkin lymphoma (all cancers excluded)	5	IVW	0.99 (0.75–1.31)	.97
–	–	–	MR-Egger	0.71 (0.36–1.41)	.4
–	–	–	Weighted median	0.93 (0.67–1.28)	.64
–	–	–	Weighted mode	0.94 (0.64–1.37)	.77
	Diffuse large B-cell lymphoma (all cancers excluded)	5	IVW	0.94 (0.54–1.64)	.84
–	–	–	MR-Egger	0.34 (0.11–1.02)	.15
–	–	–	Weighted median	0.76 (0.42–1.38)	.36
–	–	–	Weighted mode	0.67 (0.34–1.31)	.31
	Follicular lymphoma (all cancers excluded)	5	IVW	1.02 (0.69–1.52)	.91
–	–	–	MR-Egger	0.52 (0.24–1.13)	.2
–	–	–	Weighted median	0.88 (0.62–1.26)	.49
–	–	–	Weighted mode	0.84 (0.58–1.23)	.43
–	Lymphoid leukaemia	5	IVW	1.24 (0.97–1.59)	.09
–	–	–	MR-Egger	1.28 (0.69–2.38)	.49
–	–	–	Weighted median	1.15 (0.83–1.58)	.40
–	–	–	Weighted mode	1.11 (0.76–1.61)	.62
–	Mantle cell lymphoma	5	IVW	1.17 (0.75–1.81)	.50
–	–	–	MR-Egger	0.8 (0.25–2.51)	.72
–	–	–	Weighted median	1.2 (0.69–2.09)	.51
–	–	–	Weighted mode	1.28 (0.66–2.49)	.50
–	Hodgkin lymphoma (controls excluding all cancers)	5	IVW	1.21 (0.96–1.53)	.11
–	–	–	MR-Egger	1.7 (1.01–2.89)	.14
–	–	–	Weighted median	1.12 (0.84–1.48)	.44
–	–	–	Weighted mode	1.09 (0.76–1.57)	.67

DLBL = diffuse large B-cell lymphoma (all cancers excluded), FL = follicular lymphoma (all cancers excluded), HL = Hodgkin lymphoma (controls excluding all cancers), IVW = inverse-variance weighted, LL = lymphoid leukaemia, MCL = Mantle cell lymphoma, MR = Mendelian randomization, Other = other and unspecified types of non-Hodgkin lymphoma (all cancers excluded).

### 3.3. Sensitivity analysis

**Figure 4.**
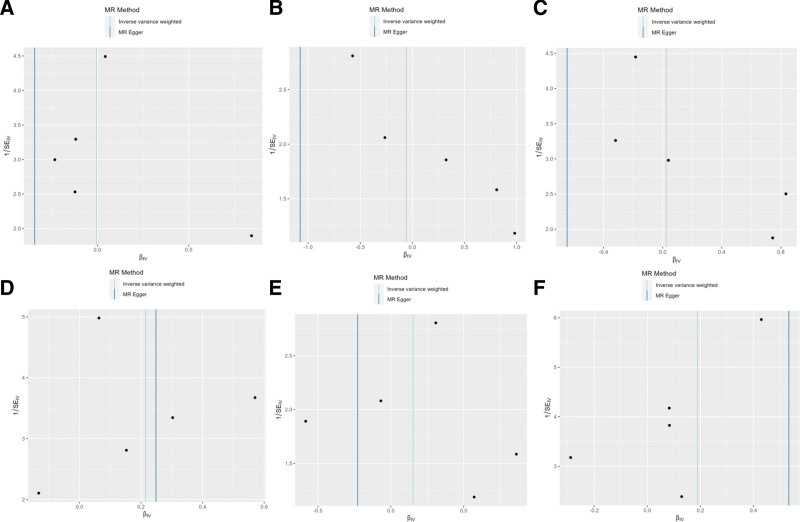
Funnel plot visualizing the outliers found by MR-Egger study, for: (A) other and unspecified types of non-Hodgkin lymphoma (all cancers excluded); (B) diffuse large B-cell lymphoma (all cancers excluded); (C) follicular lymphoma (all cancers excluded); (D) lymphoid leukaemia; (E) Mantle cell lymphoma; (F) Hodgkin lymphoma (controls excluding all cancers).

**Figure 5.**
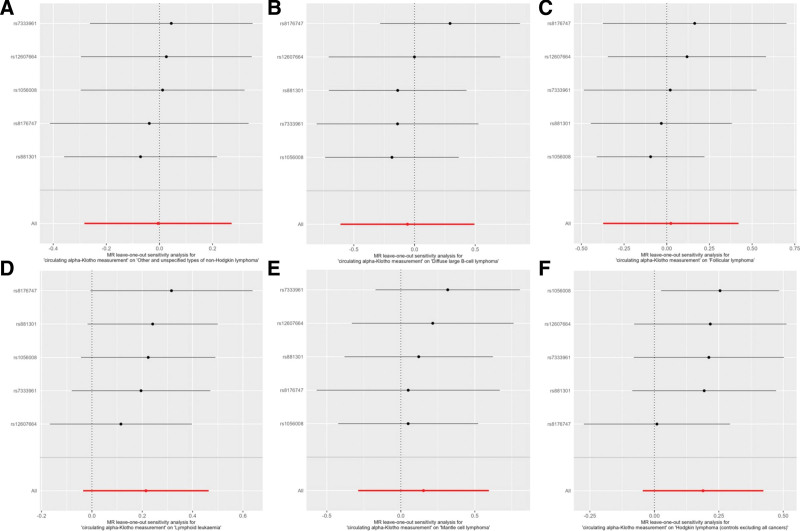
Leave-one-out sensitivity analysis of Klotho levels on B-cell lymphoma. (A) Other and unspecified types of non-Hodgkin lymphoma (all cancers excluded); (B) diffuse large B-cell lymphoma (all cancers excluded); (C) follicular lymphoma (all cancers excluded); (D) lymphoid leukaemia; (E) Mantle cell lymphoma; (F) Hodgkin lymphoma (controls excluding all cancers).

Sensitivity analyses were conducted across several parameters to evaluate the robustness of the MR findings, confirming the reliability of the results. Cochran *Q* test revealed a degree of heterogeneity, particularly evident when examining the effect on follicular lymphoma; however, this did not alter the overall null conclusion. The MR-Egger regression played a crucial role in confirming the absence of horizontal pleiotropy, supporting the validity of the causal estimates (Table 2 and Fig. 4). Additionally, the MR-PRESSO analysis did not detect any outliers, which further underpins the robustness of the findings. Finally, the leave-one-out analysis showed that no single SNP significantly drove the observed associations, indicating that the conclusions are not overly reliant on any specific genetic variant (Fig. 5). These comprehensive sensitivity checks ensure that the MR conclusions are well-founded and not subject to specific biases or anomalies. These sensitivity analyses reinforce the primary MR results, suggesting that the findings are reliable and robust against various forms of potential biases.

**Table 2:** Horizontal pleiotropy and heterogeneity test.

Exposure	Outcome	Heterogeneity		Pleiotropy	
		*Q* statistic (IVW)	*P*-value	MR-Egger Intercept	*P*-value
α-Klotho	Other	3.338	.503	0.089	.367
	DLBL	6.172	.187	0.269	.141
	FL	8.04	.09	0.177	.159
	LL	2.929	.57	−0.009	.914
	MCL	3.859	.425	0.1	.528
	HL	4.75	.314	−0.092	.256

DLBL = diffuse large B-cell lymphoma (all cancers excluded), FL = follicular lymphoma (all cancers excluded), HL = Hodgkin lymphoma (controls excluding all cancers), IVW = inverse-variance weighted, LL = lymphoid leukaemia, MCL = Mantle cell lymphoma, MR = Mendelian randomization, Other = other and unspecified types of non-Hodgkin lymphoma (all cancers excluded).

## 4. Discussion

While previous studies have extensively documented the role of the Klotho protein as a potential tumor suppressor and modulator of insulin-like growth factor 1 receptor (IGF1R) signaling in various cancers, including T- and B-cell lymphomas.^[9,10]^ However, in our study, we found no statistically significant evidence to support a causal effect of the Klotho protein on B-cell lymphoma.

Klotho function as an antiaging protein has been implicated in several types of cancer, notably renal cell carcinoma, gastric cancer, breast cancer, and colorectal cancer. In these malignancies, lower expression of Klotho often correlates with poorer prognosis, increased tumor aggression, and reduced survival rates. For instance, in renal cell carcinoma, Klotho is known to suppress tumor proliferation and promote apoptosis, while in gastric cancer, its reduced expression is linked with enhanced tumor migration and invasion.^[31–33]^ Similarly, in breast and colorectal cancers, Klotho influence extends to regulating critical signaling pathways such as the Wnt pathway, emphasizing its broad regulatory capabilities in cellular processes including proliferation, apoptosis, and the epithelial-mesenchymal transition.^[34,35]^

However, the role of Klotho in hematological malignancies like B-cell lymphoma appears less definitive. Despite earlier studies suggesting potential suppressive effects of Klotho on lymphoma progression,^[10]^ B-cell lymphoma cohort analysis using GWAS data from the FINN Large Cohort did not support a significant role for Klotho in influencing B-cell lymphoma risk. This finding may reflect a more limited or context-dependent role of Klotho in the pathogenesis of hematological cancers compared to solid tumors. Alternatively, the presence of other overriding or confounding biological factors might obscure Klotho effects in B-cell lymphoma.

The clinical implications of our study are significant as they suggest that Klotho may not universally serve as an effective biomarker or therapeutic target across all cancer types. Understanding the specific contexts and mechanisms through which Klotho influences different cancers could guide more targeted therapeutic approaches and prognostic assessments. For instance, while enhancing Klotho expression might benefit therapies in certain solid tumors, its relevance in the treatment or prevention of B-cell lymphoma remains uncertain.^[36,37]^

Although MR provides a powerful framework for inferring causality, several inherent methodological limitations should be acknowledged. First, our analysis was based on summary-level data from publicly available GWASs, which may introduce measurement error or heterogeneity due to differences in study design, phenotype definitions, or population characteristics. Second, the statistical power of MR analyses is limited when the true causal effect size is small; thus, a null finding may reflect insufficient power rather than the absence of a causal relationship. Third, the present study assumes a linear relationship between circulating Klotho levels and B-cell lymphoma risk; if the true association is nonlinear or threshold-dependent, our methods may not capture it.

## 5. Conclusion

Our results did not reveal a causal relationship between Klotho protein levels and the risk of B-cell lymphoma. To build upon our findings, future research should employ complementary approaches. First, in vitro studies using B-cell lymphoma cell lines are needed to investigate potential context-dependent biological roles of Klotho, particularly its impact on key signaling pathways like PI3K/AKT and NF-kB. Second, replication of our MR analysis in diverse, non-European populations is essential to confirm the generalizability of our null finding. Finally, prospective longitudinal studies and multi-omic analyses could clarify more complex or indirect relationships between Klotho biology and lymphomagenesis that are not captured by the current MR framework.

## Author contributions

**Data curation:** Zi Wang, Su Mao, Daobin Zhou, Wei Zhang.

**Formal analysis:** Zi Wang, Su Mao, Daobin Zhou, Wei Zhang.

## Supplementary Material




