# Supplementary material for: Causal association between circulating Klotho levels and B-cell lymphoma: A 2-sample Mendelian randomization study
Source: Medicine (Baltimore). 2025 Oct 31;104(44):e44963. doi: 10.1097/MD.0000000000044963 (PMC12582691; doi:10.1097/MD.0000000000044963)
Supplement: Supplementary file 1 [file medi-104-e44963-s001.docx]

**Supplemental Digital Content Table 1. GWAS data for B-cell lymphomas and the level of α-Klotho in circulating plasma**

| Trait | Diseases | GWAS ID | Sample size (case/control) | SNPs number |
| --- | --- | --- | --- | --- |
| Other and unspecified types of non-Hodgkin lymphoma (all cancers excluded) | non-Hodgkin's lymphoma | finn-b-CD2_NONHODGKIN_NAS_EXALLC | 533/180,756 | 16,380,337 |
| Diffuse large B-cell lymphoma (all cancers excluded) | Diffuse large B-cell lymphoma | finn-b-C3_DLBCL_EXALLC | 209/174066 | 16,380,443 |
| Follicular lymphoma (all cancers excluded) | Follicular lymphoma | finn-b-CD2_FOLLICULAR_LYMPHOMA_EXALLC | 522/180,756 | 16,380,337 |
| Lymphoid leukemia | Lymphoid leukemia | finn-b-CD2_LYMPHOID_LEUKAEMIA | 663/218,129 | 16,380,466 |
| Mantle cell lymphoma | Mantle cell lymphoma | C3_MANTLE_CELL_LYMPHOMA_EXALLC | 210/314193 |  |
| Hodgkin lymphoma (controls excluding all cancers) | Hodgkin lymphoma | CD2_HODGKIN_LYMPHOMA_EXALLC | 846/324650 |  |
| circulating alpha-Klotho measurement | The level of α-Klotho in circulating plasma | GCST90091247 | 4,675 | 4,675 |
